# A Self-management SMS Text Messaging Intervention for People With Inflammatory Bowel Disease: Feasibility and Acceptability Study

**DOI:** 10.2196/34960

**Published:** 2022-05-06

**Authors:** Jacob A Rohde, Edwin B Fisher, Marcella H Boynton, Deen Freelon, Dennis O Frohlich, Edward L Barnes, Seth M Noar

**Affiliations:** 1 Hussman School of Journalism and Media University of North Carolina at Chapel Hill Chapel Hill, NC United States; 2 Department of Health Behavior University of North Carolina at Chapel Hill Chapel Hill, NC United States; 3 North Carolina Translational and Clinical Sciences Institute University of North Carolina at Chapel Hill Chapel Hill, NC United States; 4 Department of Media and Journalism Bloomsburg University of Pennsylvania Bloomsburg, PA United States; 5 Division of Gastroenterology and Hepatology University of North Carolina at Chapel Hill Chapel Hill, NC United States

**Keywords:** inflammatory bowel disease, mHealth, self-management, SMS text messaging, mobile phone

## Abstract

**Background:**

Mobile health technologies can be useful for providing disease self-management information and support to people with inflammatory bowel disease (IBD).

**Objective:**

The aim of this study was to test a self-management SMS text messaging intervention for people with IBD. Our goal was to examine intervention feasibility, acceptability, and engagement and to preliminarily evaluate improvements in certain self-reported health outcomes among participants.

**Methods:**

We developed an SMS text messaging program called *Text4IBD*. The program sent daily support messages and resources about disease self-management over the course of a 2-week, single-group, pretest-posttest intervention to participants (N=114) diagnosed with IBD. We examined intervention feasibility, acceptability, and engagement through *Text4IBD* message topic recall and use of resources (ie, visiting supplemental websites recommended by the *Text4IBD* program). We also assessed pretest-posttest measures of IBD-related distress, self-efficacy, perceived support, use of coping strategies, and medication adherence. Analyses examined participants’ evaluations of the intervention and compared pretest-posttest changes in secondary outcomes using paired-samples statistics.

**Results:**

Approximately all participants who completed the intervention (n=105) were receptive to *Text4IBD* and viewed the program as feasible and acceptable. In addition, most participants (103/105, 98.1%) recalled at least one of the message topics sent by the program, and 79% (83/105) of them self-reported engaging with at least one of the external self-management resources recommended by the *Text4IBD* program. Pretest-posttest results showed reduced IBD-related distress (mean 3.33, SD 0.68 vs mean 2.86, SD 0.73; *P*<.001) and improvements in most other secondary outcomes.

**Conclusions:**

Findings from this study highlight the value of SMS text messaging as a useful digital medium for providing support to people with IBD, particularly to those who may struggle with disease-related distress. *Text4IBD* was highly feasible and acceptable and may help people self-manage their IBD. Future studies should aim to evaluate this program in a randomized controlled trial in clinical settings.

## Introduction

### Background

Inflammatory bowel disease (IBD) represents chronic gastrointestinal diseases—including Crohn disease (CD) and ulcerative colitis (UC)—that affect >3 million adults in the United States [1]. Examples of disease symptoms include chronic diarrhea, abdominal and joint pain, and fatigue. The relapsing-remitting nature of CD and UC can make self-management of disease symptoms difficult, which could increase the risk of experiencing disease-related distress. Recent evidence suggests that approximately 30% of people with IBD report symptoms of anxiety or depression [2,3], which is approximately triple that of US adults [4]. If unaddressed, such distress can lead to other harmful outcomes, such as a worse disease course and increased disease activity [5-11]. Thus, research should prioritize investigating methods to promote IBD self-management among individuals in distress.

SMS text messaging can be a useful medium to provide support. Research shows that SMS text messaging interventions have been successful in modifying health outcomes, particularly among people with chronic disease [12-14]. However, so far, few studies have investigated the effects of SMS text messaging among people with IBD. Recently, Riaz and Nielsen [15] developed a single-group pilot intervention that sent tailored SMS text messages about IBD medication and treatment to people with IBD. At the 12-week follow-up, participants increased their medication adherence and decreased their concerns about IBD treatment compared with baseline. Another study by Miloh et al [16] randomly assigned adolescents with IBD to either a medication reminder SMS text messaging intervention or a standard care control. Those in the SMS text messaging trial arm showed significant improvements in their medication adherence at the 6- and 12-month follow-ups than those in the control group.

However, not all IBD SMS text messaging studies have been effective. A randomized controlled trial tested educational SMS text messages about IBD self-management compared with standard care [17,18]. At the 1-year follow-up, there were no differences across trial arms in depressive symptoms, self-efficacy, or other outcomes such as quality of life. These null findings could be attributed to low intervention dose, as participants received messages only once or twice weekly. Another explanation is that the participants were assessed 6 months after beginning the trial, and message effects may have decreased before the assessment of the study outcomes.

These inconsistent findings indicate that more studies are needed to investigate effective applications of SMS text messaging as an intervention medium for facilitating disease self-management and support. Moreover, the current literature in this area is relatively homogenous, with many studies focusing on sending messages aimed at modifying medication adherence. Few interventions have sought to test whether such messaging efforts could impact other important outcomes, such as coping strategies or perceived support. Testing systematically designed messages that provide information about a multitude of disease self-management behaviors (beyond medication reminders) could have important implications for future efforts aimed at improving health and well-being among people with IBD.

### Objective

This formative study sought to develop and preliminarily evaluate the results of a single-group, pretest-posttest mobile health (mHealth) intervention called *Text4IBD*. This intervention provided information and support about disease self-management to people with IBD for 2 weeks via SMS text messaging. Our primary goal was to assess intervention feasibility and acceptability. On the basis of dissemination and implementation literature [19], we defined feasibility as relating to the trialability and practicability of the program as evaluated by participants in this intervention setting. We defined acceptability as participants’ perceived advantage of using the intervention, such as whether they viewed the aspects of *Text4IBD* as satisfactory. We also examined engagement with *Text4IBD* through aided message topic recall and use of resource links that were featured in the program. The secondary goal of this study was to examine changes in pretest-posttest outcomes targeted by intervention messages, such as disease-related distress and perceived support.

## Methods

### Participant Recruitment

We built a semiautomated program using Python (version 3.8.1) to identify prospective participants on Reddit and Twitter, which are 2 popular social media platforms used for IBD discourse [20]. This program actively scanned public posts in real time to flag prospective participants. Criteria for study eligibility were that posts had to (1) discuss IBD and distress and (2) be published by individuals and not research organizations, medical providers, or patient advocate accounts. We controlled for these parameters using both Reddit and Twitter stream features and human validation. Recruitment began in December 2020 and occurred on a rolling basis for approximately 6 weeks. Users who met the eligibility criteria were contacted and sent information about the *Text4IBD* program. Those interested in the intervention were instructed to click a link directing them to a screener survey. Refer to Multimedia Appendix 1 for participant identification and recruitment procedures.

### Inclusion Criteria

Inclusion criteria (assessed during the screener survey) required that participants (1) be diagnosed with IBD, (2) live in the United States, (3) have a smartphone that can receive SMS text messages, and (4) self-report experiencing IBD-related distress. For the last criterion, participants completed a 6-item measure of IBD-related distress, adapted from an existing short-form diabetes distress scale [21,22]. We chose to use and adapt this scale because it captures broad constructs associated with perceived distress and disease self-management, which can be applied to IBD. We also tested this adapted scale in a pilot study of people with IBD and found it to have high internal reliability. The scale began with the prompt, “During the past two weeks, how much have you...” and example scale items were, “felt that you are often failing with your IBD routine” and “felt discouraged to keep up with managing your IBD.” IBD-related distress was assessed on a 5-point scale ranging from “not at all” (score=1) to “a great deal” (score=5). To qualify for the study, participants needed to score above a mean of 2 (“a little”) out of 5 on the composite distress scale. Reliability of the distress scale among those who completed the screener survey was high (*α*=.85).

**Figure 1:**
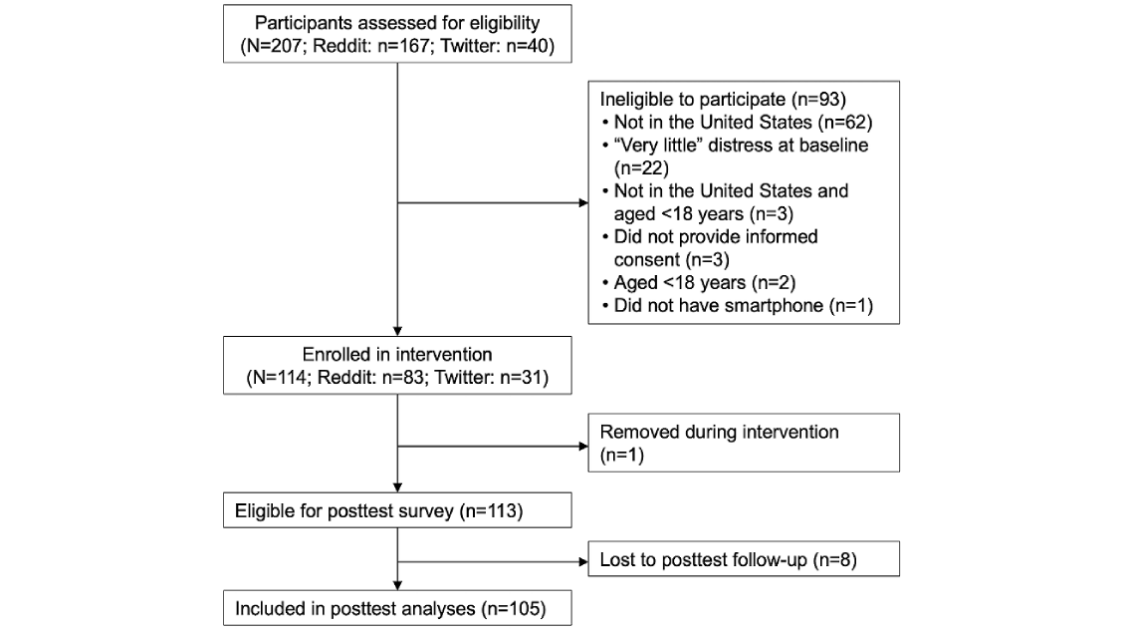
Flow diagram of participant recruitment, enrollment, and retention.

After passing the screening criteria, interested participants provided informed consent and continued to the pretest survey. In total, 207 individuals were screened for eligibility. Of these 207 individuals, 114 (55.1%) individuals enrolled in the intervention. Of these 114 participants, 1 (0.9%) participant was removed because the intervention SMS text messaging system was unable to send messages to the phone number provided to the researchers and 8 (7%) participants were lost to posttest follow-up, resulting in 105 (92.1%) participants completing the study (Figure 1).

### Intervention Design

We programmed *Text4IBD* to send daily support messages about IBD self-management to the participants’ smartphones for 2 weeks. The topics of the support messages varied but centered around three self-management domains: (1) physical IBD symptoms, (2) IBD and mental health, and (3) IBD and nutrition. The first and second domains each contained 5 support messages, and the third domain contained 4 support messages (14 unique messages in total).

Support messages were constructed in 2 parts (ie, a message component pair). The first component acknowledged and validated a difficult aspect of IBD self-management. The second offered advice addressing the struggle in the first message component. For clarity, we refer to each message component pair as a singular *support message*. The advice offered by the support messages was informed by public content from IBD research organizations (eg, Crohn’s and Colitis Foundation) to ensure that the messages were based on scientific and expert opinions. A larger set of messages was pilot-tested in a web-based study of 44 individuals with IBD. The 14 messages in this study were chosen and refined from that larger set.

Several support messages also contained links to web-based resources from the same research organizations. The purpose of these resources was to provide additional information about self-management topics beyond what was discussed in the support messages. Resource links were in the form of a customized Bitly URL attached at the end of support messages (eg, “For more information, check out this link: [URL here]”). All support messages were reviewed for accuracy and credibility by one of the authors (EB), a gastroenterologist who specializes in IBD treatment.

**Figure 2:**
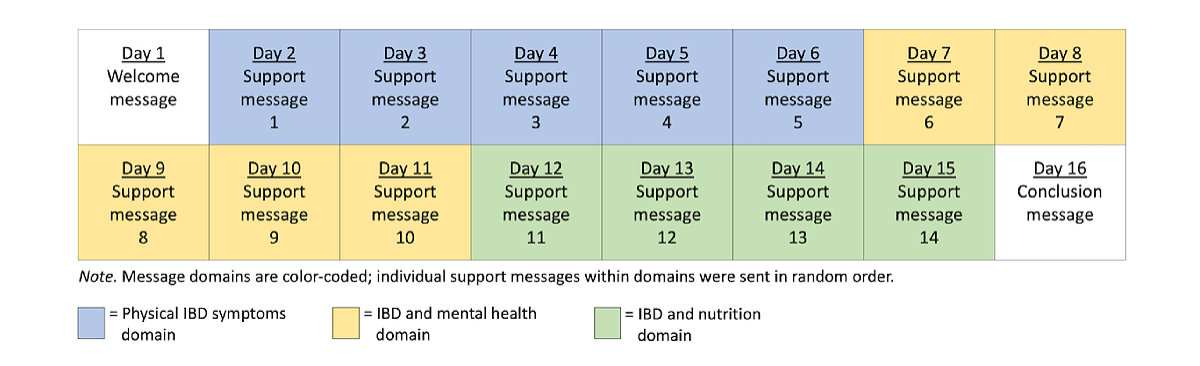
SMS text messaging schedule for the *Text4IBD* program. IBD: inflammatory bowel disease.

**Figure 3:**
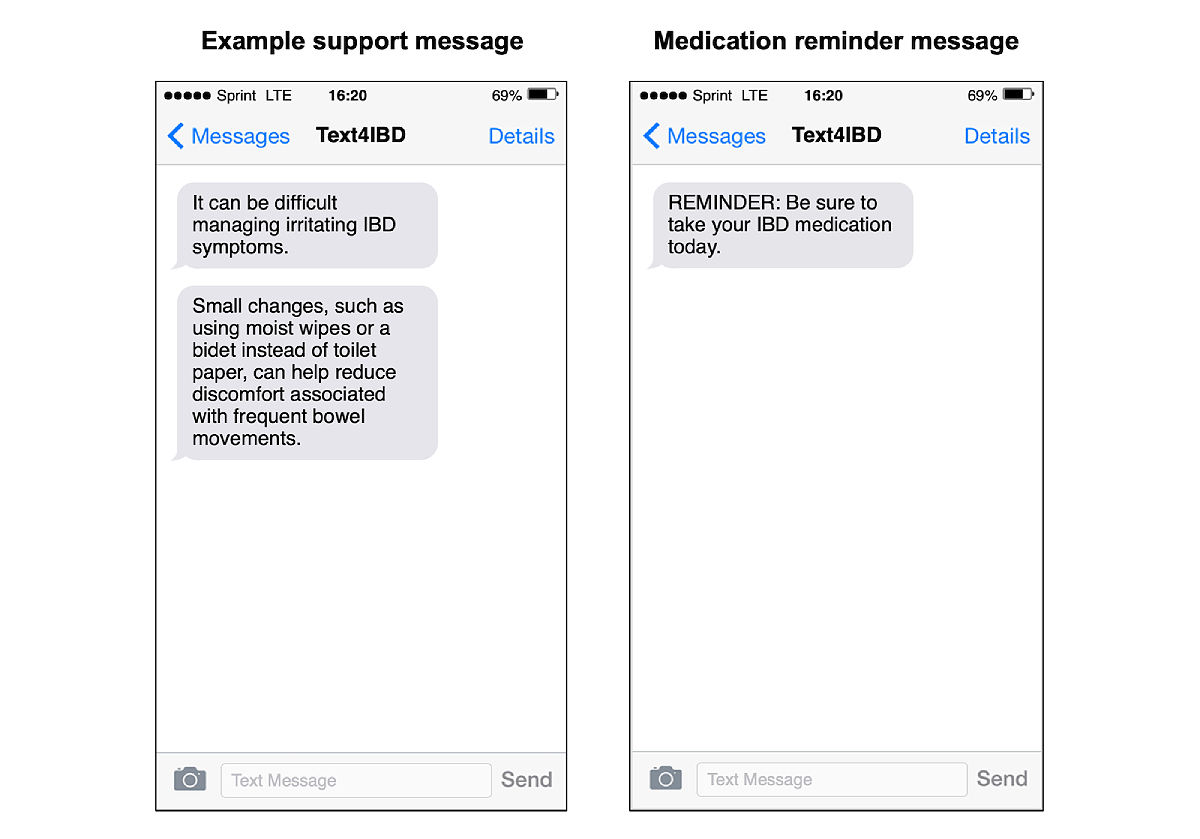
Example SMS text messages sent by the *Text4IBD* program. IBD: inflammatory bowel disease.

*Text4IBD* sent support messages to the participants’ phones at their preferred time of the day. The order in which the messages were sent was based on a partially static schedule (Figure 2). Participants first received all messages from the *physical IBD symptoms* domain, followed by all messages from the *IBD and mental health* and, then, from the *IBD and nutrition* domains; however, the order in which the support messages were sent within each domain block was randomized. *Text4IBD* also sent daily medication reminders to participants who reported currently taking daily oral medication in the pretest survey. These messages stated, “REMINDER: Be sure to take your IBD medication today.” Similar to the support messages, participants indicated a preferred time they wanted to receive their reminder message. Refer to Figure 3 for examples of *Text4IBD* messages and Multimedia Appendix 2 for all the support messages.

### Procedure

This study featured a 16-day intervention period. First, the participants completed the screener and pretest surveys (day 0) and, then, were enrolled in *Text4IBD*. After enrollment (day 1), participants received a message welcoming them to the study. Starting on day 2, participants received support messages each day for 14 days. Finally, participants received a last message (day 16) informing them that the *Text4IBD* program was complete and that they should receive an email containing a link to take a final survey within 24 hours. Participants were eligible to earn an Amazon gift card worth up to US $40 for participating (US $20 for completing each of the pretest and posttest surveys). Participants received their incentive by the end of the month in which they completed the study. Similarly, those who completed only the pretest survey received their incentive by the end of the month.

### Ethics Approval

The University of North Carolina at Chapel Hill Institutional Review Board approved all the study procedures (20-2201).

### Measures

#### Participant Demographics and IBD Characteristics

For demographics, the pretest survey assessed participants’ age, gender, race, Hispanic ethnicity, education, household income, and sexual orientation. For IBD characteristics, the pretest survey assessed the type of IBD that the participants had (UC, CD, indeterminate colitis, or other), the age at which they were diagnosed with IBD, and whether they were currently taking daily oral medication to treat their IBD or maintain IBD symptoms. The pretest survey also asked participants to rate their disease activity over the past 6 months using the single-item Manitoba IBD Index [23].

#### Intervention Feasibility

The posttest survey asked participants how difficult or easy they found participating in the study to be, whether they would enroll in the study again, and whether they would recommend the study to someone else with IBD (all on 5-point scales with neutral middle points). Feasibility items were dichotomized for analysis, such that responses above the middle point (ie, score of 4 or 5) were categorized as perceiving that component of the intervention as feasible. Participants were also asked what they thought about the frequency of messages they received during the study (with responses being “too little,” “about right,” or “too much”) and how many of those messages they read (5-point scale ranging from “none” to “all of them”). Feasibility items were informed by implementation and dissemination literature [19].

#### Intervention Acceptability

The posttest survey asked participants about their overall satisfaction with the content of the messages and resources sent by *Text4IBD* (5-point scales ranging from “not at all” to “extremely”). Those who were currently taking daily oral medication were asked how useful they thought the reminder messages were (5-point scale ranging from “not at all” to “extremely”). Participants also answered questions about their attitudes toward the study. Attitudes were assessed using a 4-item scale developed for this study. The scale began with the prompt, “Overall, would you say participating in this study was...” Responses to attitude items were on 10-point bipolar scales. Response anchors were (1) not helpful–helpful, (2) not informative–informative, (3) not supportive–supportive, and (4) not useful–useful. Reliability of the scale was high (*α*=.94). Acceptability items were informed by implementation and dissemination literature [19].

#### Aided Topic Recall

The posttest survey asked participants to select message topics (all the applicable topics) that they remembered being sent by the *Text4IBD* program. Topics were the following: (1) IBD and mental health, (2) IBD nutrition, (3) IBD diets, (4) IBD management, (5) exercise and stress, (6) IBD symptom tracking, and (7) food journaling. In addition, participants could choose 3 topics in the same list that were not part of the *Text4IBD* program. These foil topics were the following: (1) irritable bowel syndrome versus IBD symptoms, (2) IBD procedures, and (3) IBD and ostomies. Participants could also choose that they did not recall seeing any of these topics.

#### Use of Linked Resources

The posttest survey asked participants to select the linked resources (all the applicable resources) that they clicked during the intervention. This measure was to evaluate whether participants opened the messages on their phones, as it was not possible to collect those data. Resources in this list were the following: (1) MyGut, (2) IBD nutrition and what to eat, (3) coping strategies to improve mental health, (4) special IBD diets, (5) COVID-19 and mental health, (6) web-based IBD communities, and (7) IBD expert questions and answers.

#### Pretest-Posttest Outcomes

Both pretest and posttest surveys assessed measures of IBD-related distress [21,22], perceived IBD support (developed for this study), self-efficacy [24], use of 3 different coping strategies [25], and medication adherence [26] as secondary outcomes. Self-efficacy assessment included four subscales: (1) stress and emotions management, (2) medication management, (3) symptoms management, and (4) remission management. Medication adherence was reported as a binary outcome (complete adherence vs not complete adherence), and all other secondary outcomes were reported as approximations of continuous measures from Likert-style scales. Refer to Multimedia Appendix 3 [21,22,24-26] for all the items associated with secondary outcomes.

### Data Analysis

We used descriptive statistics to characterize intervention feasibility, acceptability, and engagement (ie, aided topic recall and use of linked resources). We used 2-tailed paired-samples *t* tests and McNemar tests to examine pretest-posttest changes in the secondary outcomes for continuous and categorical variables, respectively. Only the participants who completed both surveys were included in this analysis. We calculated effect size estimates for paired-sample comparisons of continuous variables using Cohen *d*. We interpreted effect sizes of Cohen *d*=0.2 as small, Cohen *d=*0.5 as medium, and Cohen *d* ≥0.8 as large. Analyses were performed using R (version 3.6.2; R Foundation for Statistical Computing).

## Results

### Participant Demographics and IBD Characteristics Obtained From the Pretest Survey

Mean age of the participants was 29 years (range 19-53 years; Table 1); most participants were White (95/114, 83.3%), and approximately 8.8% (10/114) of the participants identified as Hispanic. More than half (68/114, 59.6%) of the participants were female, and more than one-third (44/114, 38.6%) identified as gay, lesbian, or bisexual. Slightly less than half (52/114, 45.6%) of the participants had a Bachelor’s degree or higher. Household income varied, with 46.5% (53/114) of the participants earning <US $50,000; 35.1% (40/114) earning between US $50,000 and US $99,999; and 16.7% (19/114) earning ≥US $100,000.

**Table 1:** Demographic and IBD^a^ characteristics of the study sample obtained from the pretest survey (N=114).

			Values	
Age (years), mean (SD; range)			29.11 (7.28; 19-53)	
**Gender, n (%)**				
	Female	68 (59.6)		
	Male	36 (31.6)		
	Other or prefer not to say	10 (8.8)		
**Race, n (%)**				
	White	95 (83.3)		
	Black or African American			2 (1.8)
	Asian			2 (1.8)
	Other or multiracial	15 (13.2)		
Hispanic, n (%)			10 (8.8)	
Gay, lesbian, or bisexual, n (%)			44 (38.6)	
**Education, n (%)**				
	High school or less	12 (10.5)		
	Some college or associate degree	50 (43.9)		
	Bachelor’s degree	40 (35.1)		
	Graduate or professional degree	12 (10.5)		
**Annual household income (US $), n (%)**				
	0-29,999	30 (26.3)		
	30,000-49,999	23 (20.2)		
	50,000-79,999	24 (21.1)		
	80,000-99,999	16 (14)		
	≥100,000	19 (16.7)		
	No response	2 (1.8)		
**IBD type, n (%)**				
	Crohn disease	83 (72.8)		
	Ulcerative colitis	22 (19.3)		
	Other	9 (7.9)		
Age at diagnosis (years), mean (SD)			22.32 (8.32)	
**Years since diagnosis, mean (SD)**			6.80 (6.44)	
	≤1, n (%)	31 (27.2)		
	2-5, n (%)	28 (24.6)		
	>5, n (%)	55 (48.2)		
**IBD activity,^b^ mean (SD)**			4.68 (1.28)	
	Remission, n (%)	36 (31.6)		
	Rarely active, n (%)	34 (29.8)		
	Occasionally active, n (%)	27 (23.7)		
	Sometimes active, n (%)	8 (7)		
	Often active, n (%)	6 (5.3)		
	Constantly active, n (%)	3 (2.6)		
Complete medication adherence^c^ (n=73), n (%)			41 (56)	

^a^IBD: inflammatory bowel disease.

^b^IBD activity based on the Manitoba IBD Index, where high values indicate worse disease activity.

^c^Assessed only among those who reported taking daily oral IBD medication in the pretest survey.

Most participants had CD (83/114, 72.8%) or UC (22/114, 19.3%); however, a small proportion (9/114, 7.9%) self-reported other IBD diagnoses such as lymphocytic or collagenous colitis. Mean age at diagnosis was 22.32 (SD 8.32) years, and approximately half (59/114, 51.8%) of the participants had been living with IBD for ≤5 years. In all, 31.6% (36/114) of the participants reported being in disease remission the past 6 months, whereas only 7.9% (9/114) reported active IBD symptoms (ie, experiencing symptoms “often” or “constantly”) over the same time. Most participants (73/114, 64%) reported currently taking daily oral IBD medication. Approximately half (41/73, 56%) of them reported complete medication adherence.

### Feasibility and Acceptability

Approximately all participants (101/105, 96.2%; Table 2) said it was easy to participate in the study, that they would participate again if given the option (98/105, 93.3%), and that they would recommend the study to others with IBD (99/105, 94.3%). Most participants (90/105, 85.7%) thought that the message frequency during the intervention was approximately right, and 91.4% (96/105) of the participants reported reading “all” or “a lot” of the messages. The perceived usefulness of the medication reminder messages was modest (mean 3.37, SD 1.39). In addition, overall attitudes toward *Text4IBD* were positive (mean 8.03, SD 2.04), and participants tended to be satisfied with the content of the support messages (mean 3.81, SD 1).

**Table 2:** *Text4IBD* feasibility, acceptability, and engagement (n=105).

		Values
Easy to participate, n (%)		101 (96.2)
Would participate again, n (%)		98 (93.3)
Would recommend to others with IBD,^a^ n (%)		99 (94.3)
Message frequency approximately right, n (%)		90 (85.7)
**Number of messages read, n (%)**		
	All of them	76 (72.4)
	A lot	20 (19)
	Some	8 (7.6)
	Very few	1 (0.9)
**Overall attitude toward the intervention, mean (SD)**		8.03 (2.04)
	Helpful	7.88 (2.29)
	Informative	7.99 (2.24)
	Supportive	8.33 (2.05)
	Useful	7.91 (2.34)
Medication reminder was useful,^b^ mean (SD)		3.37 (1.39)
Satisfied with message content, mean (SD)		3.81 (1)
Satisfied with message resources,^c^ mean (SD)		3.64 (0.98)
Used at least one message resource, n (%)		83 (79)
**Message resources accessed,^d^ n (%)**		
	MyGut	45 (42.9)
	IBD nutrition and what to eat	45 (42.9)
	Coping strategies to improve mental health	41 (39)
	Special IBD diets	39 (37.1)
	COVID-19 and mental health	38 (36.2)
	Web-based IBD community	32 (30.5)
	IBD expert questions and answers	24 (22.9)

^a^IBD: inflammatory bowel disease.

^b^Assessed only among those who reported taking daily oral IBD medication in the posttest survey (n=65).

^c^Assessed only among those who reported accessing at least one message resource (n=83).

^d^Variables are not mutually exclusive.

### Engagement With Message Topics and Use of Linked Resources

**Figure 4:**
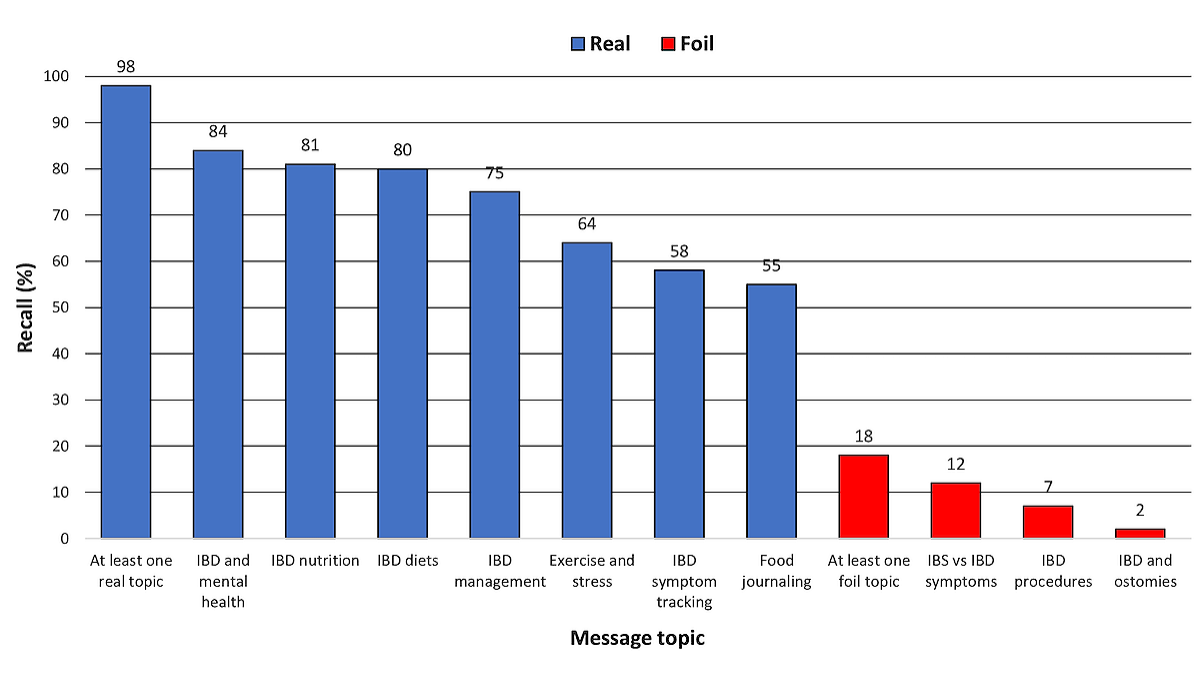
Aided topic recall of the *Text4IBD* messages. IBD: inflammatory bowel disease; IBS: irritable bowel syndrome.

In the posttest survey, approximately all participants (103/105, 98.1%; Figure 4) reported recalling at least one *Text4IBD* message topic. The most recalled topic was IBD and mental health (84/105, 80%), followed by IBD and nutrition (81/105, 77.1%), IBD diets, (80/105, 76.2%), and IBD management (75/105, 71.4%). The least recalled topics were IBD symptom tracking (58/105, 55.2%) and food journaling (55/105, 52.4%). A modest number of participants (19/105, 18.1%) falsely reported recalling one or more of the 3 foil topics, with the most selected foil topic (irritable bowel syndrome vs IBD symptoms) being inaccurately recalled by 12.4% (13/105) of the participants.

In the posttest survey, 79% (83/105; Table 2) participants self-reported using at least one *Text4IBD* linked resource. Linked resource satisfaction was modest (mean 3.64, SD 0.98). The most used resources were MyGut (an IBD self-management mobile app; 45/105, 42.9%), IBD nutrition and what to eat (45/105, 42.9%), coping strategies to improve mental health (41/105, 39%), special IBD diets (39/105, 37.1%), and COVID-19 and mental health (38/105, 36.2%). The web-based IBD communities (32/105, 30.5%) and IBD expert questions and answers (24/105, 22.9%) resources were accessed by few participants.

### Differences Between Pretest and Posttest Outcomes

In the posttest survey, participants reported lower IBD-related distress than during the pretest survey (*P*<.001; Table 3). This change constitutes a medium to large effect size (Cohen *d*=0.77). Participants reported a modest improvement in their perceived IBD support from pretest to posttest period (*P*<.001; Cohen *d*=0.47). Participants also reported pretest-posttest improvements in stress and emotions management (*P*<.001), remission management (*P*<.001), and symptoms management (*P*<.001) self-efficacy. These changes exhibited medium to large effect sizes (Cohen *d* range 0.58-0.75). Changes in the use of coping strategies varied. Participants reported minor increases in their use of relaxation (*P*=.009) and positive thinking (*P*=.03) coping strategies from pretest to posttest period. In contrast, in the posttest survey, participants reported worse scores for the altering their diet to improve IBD symptoms coping strategy than in the pretest survey (*P*=.02). Changes among the 3 coping strategies exhibited small effect sizes (Cohen *d* range –0.22 to 0.26). Finally, participants currently taking daily oral medication for their IBD did not observe any pretest-posttest improvements in medication adherence (*P*=.45) or medication management self-efficacy (*P*=.49).

**Table 3:** Pretest-posttest differences among study outcomes (n=105).^a^

		Pretest assessment	Posttest assessment	*P* value	Cohen *d*
IBD^b^-related distress, mean (SD)		3.33 (0.68)	2.86 (0.73)	<.001	0.77
Perceived IBD support, mean (SD)		2.89 (0.92)	3.26 (0.93)	<.001	0.47
**Self-efficacy, mean (SD)**					
	Remission management	4.94 (1.94)	6.39 (1.97)	<.001	0.75
	Stress and emotions management	5.19 (1.85)	6.45 (1.76)	<.001	0.69
	Symptoms management	4.21 (1.96)	5.53 (2.09)	<.001	0.58
	Medication management^c^	9.32 (2.23)	9.66 (1.64)	.49	0.09
**Coping strategies, mean (SD)**					
	Use of relaxation techniques	2.63 (1.12)	2.93 (1.07)	.009	0.26
	Think positively about IBD	2.47 (1.24)	2.69 (1.14)	.03	0.21
	Alter diet to improve IBD	3.44 (1.26)	3.18 (1.09)	.02	–0.22
Complete medication adherence^c^ (n=61), n (%)		36 (59)	40 (65.6)	.45	N/A^d^

^a^Measures were assessed on 5-point scales, except self-efficacy subscales (11-point scales) and complete medication adherence (dichotomous outcome); low values in the posttest survey for IBD-related distress indicate better outcome, whereas high values in the posttest survey for perceived IBD support, self-efficacy subscales, coping strategies, and complete medication adherence indicate a better outcome. We also ran the analyses controlling for age, gender, education level, type of IBD, time since IBD diagnosis, and disease activity and did not find any differences in the pattern of effects. Thus, we present the findings without adjustment.

^b^IBD: inflammatory bowel disease.

^c^Assessed only among those who reported taking daily oral IBD medication both in the pretest and posttest surveys.

^d^N/A: not applicable.

## Discussion

### Principal Findings

This study sought to examine *Text4IBD* feasibility, acceptability, and engagement and to preliminarily evaluate pretest-posttest changes in health outcomes targeted by the intervention. Results demonstrate that delivering an IBD support intervention by SMS text message was feasible and that participants viewed the program as acceptable. Participants also recalled most of the support message topics, and many used the linked resources. Furthermore, results showed improvements in several self-reported outcomes, including IBD-related distress and perceived support. These findings provide much-needed empirical evidence to the ongoing discussion about the utility of eHealth in personal IBD health care [27-32] and suggest that digital technologies can play an important role in self-managing IBD symptoms.

The primary goal of this study was to assess whether people with IBD would be receptive to a support-based SMS text messaging intervention, and the results were promising. Approximately all participants evaluated *Text4IBD* as feasible and acceptable across multiple aspects of the program, including satisfaction with message content and frequency. Moreover, attitudes toward *Text4IBD* were typically scored ≥8 out of 10, and approximately all participants reported that they would participate in the study again if given the opportunity. This level of satisfaction with the program indicates the value of offering support to people with IBD.

Indicators of engagement with *Text4IBD* were also positive. For example, 79% (83/105) of the participants reported accessing at least one linked resource, aided topic recall was high overall, and few participants reported recalling a foil message topic. These findings support the literature showing that people with IBD tend to be receptive to the idea of eHealth interventions for disease self-management [33-37] and that digital technologies can be used for therapeutic purposes [38-40].

A secondary goal of this study was to test whether an SMS text messaging intervention would be useful in improving health outcomes. Although the lack of a control group precludes us from making strong causal conclusions, our pretest-posttest findings provide compelling evidence that the intervention may have reduced disease-related distress. This result has important clinical implications, given the high levels of distress that people with IBD typically experience [2,3] and the various health consequences associated with distress [5-11]. Results also suggest that participants increased their perceptions of IBD support, confidence to self-manage IBD (ie, self-efficacy outcomes), and use of some coping strategies. These findings support the implementation of similar mHealth frameworks in future self-management interventions for this population.

A surprising result of this study was its null findings regarding medication-related outcomes. In contrast to other research [15,16], participants did not show any pretest-posttest improvements in medication self-efficacy or adherence outcomes. Regarding the self-efficacy outcome, this finding is likely explained by the fact that mean pretest scores were high (mean 9.32 out of 11, SD 2.23), leaving little scope for improvement. In contrast, medication adherence was modest during the pretest period (36/61, 59%) and only marginally improved by the posttest period (40/61, 66%). This nonsignificant change could be because medication reminder messages were only sent once per day, which may not help individuals taking multiple medications at different times throughout the day. Notably, participants in this study were individuals with IBD who likely had an established medication regimen. A similar type of intervention might prove more effective for those with new IBD diagnoses or those trying to implement a new regimen.

It should be stated that these null findings do not suggest that medication reminder messages have no role in IBD interventions. In fact, the opposite is true, as evidence generally shows positive effects of such interventions on disease outcomes in studies among people with chronic disease [12]. Instead, future studies should improve the reminder message component of this intervention, such as by better tailoring the messages to participants’ needs (eg, allowing participants to set medication reminders at times and frequencies that fit their schedule).

Finally, participants reported a low likelihood of altering their diet to improve IBD symptoms from the pretest to posttest period, indicating a worse outcome. A possible explanation for this could be that altering one’s diet is not a *regular* behavior. That is, people with IBD likely do not experiment with their diet, especially if they have adapted to a nutrition plan that works for them. However, diet is integral to IBD self-management, and thus, studies examining the viability of using mHealth to improve nutrition for those with IBD warrants further investigation.

Overall, findings from this formative study provide insight regarding the design, implementation, and dissemination of mHealth interventions in this area. As previously discussed, past IBD and SMS text messaging interventions have tended to last between 3 and 12 months [15-18], with follow-up results exhibiting inconsistent findings. In contrast, *Text4IBD* was intentionally designed to expose participants to a large number of diverse support messages in a short period. Our high sample retention (105/114, 92.1%) and the effects exhibited between pretest-posttest secondary outcomes suggest that interventions of this design may be well suited for delivering support about IBD self-management and for lessening participants’ burden and fatigue. Of course, it should be stated that the magnitude of our findings is attributable, in part, to the relatively short duration of this study and the assessment of outcomes immediately after intervention completion [41]. Therefore, researchers interested in examining longitudinal effects may wish to use other study designs. Nonetheless, future studies should consider iteratively testing *Text4IBD* across varying durations and populations to assess its efficacy over time.

Strengths of this study include high retention of participants at the 2-week posttest assessment, a diverse set of support messages about IBD self-management selected from pilot study findings, and implementation of a custom mHealth program that allowed participants to choose when to receive support messages. The main limitation of this study is that it used a single-group design, which limits our ability to fully attribute changes in study outcomes to the intervention. Future studies should test *Text4IBD* in a randomized controlled trial. Another limitation is that the participants were recruited via convenience sampling methods using public social media data. Factors such as digital literacy and nongeneralizable representation among social media users—as evidenced by characteristics of our study sample (ie, predominately White and female)—mean that additional studies are needed to examine whether our findings apply to all people with IBD.

### Conclusions

We developed *Text4IBD* to provide information and support about disease self-management to people with IBD. Our SMS text messaging intervention was feasible and highly acceptable, and intervention retention was high. Given that people with IBD struggle with self-managing their disease, the results from this study are encouraging and support the future use and development of digital technologies to improve health outcomes in this population.

## Appendix

Multimedia Appendix 1 Social media recruitment.

Multimedia Appendix 2 *Text4IBD* support messages.

Multimedia Appendix 3 Pretest-posttest outcomes.

## Abbreviations

CD: Crohn disease
IBD: inflammatory bowel disease
mHealth: mobile health
UC: ulcerative colitis
