# Supplementary material for: A Self-management SMS Text Messaging Intervention for People With Inflammatory Bowel Disease: Feasibility and Acceptability Study
Source: JMIR Form Res. 2022 May 6;6(5):e34960. doi: 10.2196/34960 (PMC9123538; doi:10.2196/34960)
Supplement: Multimedia Appendix 1 [file formative_v6i5e34960_app1.docx]

**Appendix 1: Social media recruitment**

Criteria for identifying prospective participants for the *Text4IBD* intervention using social media were that social media posts had to 1) contain IBD *and* distress-related content and 2) be published by individuals and not research organizations, medical providers, or patient advocate accounts. For the first criterion, we created a list of approximately 300 keywords to filter for posts discussing IBD and distress in real time. IBD keywords were “ibd,” “crohn,” “colitis,” “inflammatory bowel disease,” “ileitis,” and “ileoceceal.” These keywords represent either disease categories or the associated umbrella terminology of IBD. Example distress keywords included “stress,” “unhappy,” and “terrified.” We also included multi-word phrases in this filtering process, such as “looking for advice” and “need help.” This parameter was controlled for using Reddit and Twitter API stream features. The second criterion was that the social media posts were not published by established research organizations or by IBD advocate and support accounts. This parameter only applied to data collected from Twitter. To control for these posts, we created a “block” list of Twitter usernames from IBD-related research institutions and advocate/support profiles such as “@CrohnsColitisFn.” These usernames were filtered out during the identification process in real time.

The purpose of the above automated filtering program was to only assist in identifying potential prospective participants for the current study. Researchers evaluated all social media posts collected by the program and made the final determination if a post met study eligibility, as well as if the user should be contacted for recruitment. We tested the reliability of this classification process on a 2-week pilot sample of 1,907 tweets and 2,268 Reddit posts collected from the program. We combined both data sets and randomly extracted 10% of the posts. These posts were reviewed by individuals familiar with this study. Two human coders independently evaluated the posts and categorized each as either meeting eligibility criteria for the current study (i.e., posts published by people discussing distress-related content about their IBD) or not. Reliability of the categorical results between the two coders was acceptable ([Krippendorff’s coefficient] *α*=.76; percent agreement=92%).

During the 6-week recruitment period, we extracted the results of the previous day’s sample of identified social media posts and classified whether the users may be eligible to participate in the *Text4IBD* intervention. For the most part, if social media posts were from users discussing disease-related distress, they were categorized as eligible; however, we excluded those who expressed acute distress, such as users talking about suicide or other forms of self-harm because the *Text4IBD* program was not designed for those with acute distress. Users deemed as eligible were individually contacted and sent information about the *Text4IBD* program via a direct message (for privacy purposes) on social media platform. Those interested in the program were instructed to click on a link that directed them to an online screener survey.
