# Supplementary material for: A Self-management SMS Text Messaging Intervention for People With Inflammatory Bowel Disease: Feasibility and Acceptability Study
Source: JMIR Form Res. 2022 May 6;6(5):e34960. doi: 10.2196/34960 (PMC9123538; doi:10.2196/34960)
Supplement: Multimedia Appendix 2 [file formative_v6i5e34960_app2.docx]

**Appendix 2: Text4IBD support messages**

| **Self-management domain 1: Physical IBD symptoms** | |
| --- | --- |
| **Message 1 (validating)** | **Message 2 (advising)** |
| - It can be difficult managing irritating IBD symptoms.   Message topic: Symptom discomfort | - Small changes, such as using moist wipes or a bidet instead of toilet paper, can help reduce discomfort associated with frequent bowel movements. |
| - It can be tough getting through the day feeling bloated because of IBD.   Message topic: Bloating/gas | - Eating smaller meals and avoiding caffeine and carbonated beverages can help reduce gas and make you feel less bloated. |
| - IBD flares aren’t easy to handle.   Message topic: Flare tracking | - A good way to manage flares is to keep track of your symptom activity using an app such as “MyGut.” Being aware of symptom changes can also help doctors recommend treatment options. For info about MyGut, go here: <http://bit.ly/My_Gut> |
| - Managing IBD can take a lot of energy.   Message topic: Energy | - Building in extra time in the morning, such as by packing lunches or picking out clothes the night before, can make starting your day a little easier. |
| - It’s not always easy to get good advice about IBD symptoms.   Message topic: Advice seeking | - If you’re ever uncertain about how to manage IBD, you can use the “Expert Q&A” website, which is run by doctors trained to answer any questions you may have. You can even post questions anonymously. Check out the site here: <http://bit.ly/IBD_qa> |

| **Self-management domain 2: IBD and mental health** | |
| --- | --- |
| **Message 1 (validating)** | **Message 2 (advising)** |
| - IBD symptoms can often be unpredictable, which can cause anxiety.   Message topic: Anxiety | - Practicing simple breathing or muscle relaxation exercises can help reduce stress. For more info about IBD mental health exercises, visit this site: <http://bit.ly/IBDMentalHealth> |
| - Stress can make it difficult to manage IBD.   Message topic: exercise and stress | - Short exercises, such as going on walks or riding a bike, can improve your mental health and strengthen your immune system, which can help keep your IBD in check. |
| - Staying on top of symptoms can feel overwhelming at times.   Message topic: Discouragement | - It’s important to tackle IBD one day at a time. If you’re feeling discouraged about IBD, try setting aside some time in your day to focus on self-care, such as by doing activities that you enjoy and bring you comfort. |
| - Managing IBD during the COVID-19 pandemic can be stressful.   Message topic IBD and COVID-19 | - Staying in touch with friends and family virtually can help you cope during this pandemic. For more info about managing IBD during COVID-19, check out this site: <http://bit.ly/IBD_covid> |
| - It’s common for people with IBD to struggle with their mental health.   Message topic: Social support | - Online communities can be a useful and convenient resource for finding support from other people with IBD. Learn more about online IBD communities here: <http://bit.ly/IBD_community> |

| **Self-management domain 3: IBD and nutrition** | |
| --- | --- |
| **Message 1 (validating)** | **Message 2 (advising)** |
| - Figuring out what dietary practices work best for managing IBD can be challenging.   Message topic: General nutrition | - Avoiding processed foods (such as candy and ice cream), eating balanced meals, and staying hydrated throughout the day can help keep symptoms under control. |
| - There are a lot of IBD diets, and it can be difficult learning about all of them.   Message topic: IBD diets | - The low fiber diet eliminates certain high fiber foods, such as leafy vegetables and whole grains, which can help reduce cramping and bowel movements. For info about other IBD diets, go here: <http://bit.ly/IBDDiets> |
| - Knowing what to eat during an IBD flare isn’t always clear.   Message topic: Helpful foods | - Certain nutritious foods, such as low-fiber fruits (bananas) and lean proteins (white meat chicken), can be easier to digest when experiencing symptoms. For more tips about IBD and nutrition, check out this site: <http://bit.ly/IBDNutrition> |
| - No two people with IBD react the same to eating certain foods.   Message topic: Food journaling | - Keeping a food journal to track what you eat can help you figure out if certain foods make your symptoms worse. This can help eliminate trigger-causing foods from your diet. |
