# Supplementary material for: A Self-management SMS Text Messaging Intervention for People With Inflammatory Bowel Disease: Feasibility and Acceptability Study
Source: JMIR Form Res. 2022 May 6;6(5):e34960. doi: 10.2196/34960 (PMC9123538; doi:10.2196/34960)
Supplement: Multimedia Appendix 3 [file formative_v6i5e34960_app3.docx]

**Appendix 3: Pretest-posttest outcomes**

| **ID** | **Item** | **Response scale** |
| --- | --- | --- |
| **Medication adherence** | | |
| Medication user | Are you currently taking daily oral medication to treat your IBD? | 1. Yes  2. No |
| Medication adherence | Regarding taking your IBD medication over the past 2 weeks, how often did you forget to take it?  Source: Adapted from MARS-5 scale [26]. | 1. Never 2. A little 3. Sometimes 4. Most of the time  5. Very often  Item dichotomized for analyses (complete adherence [item 1] vs not adherent [items 2-5]).  Only asked to participants currently taking daily oral medication. |
| **IBD-related distress** | | |
| Distress prompt | In the past 2 weeks, how much have you felt…  Source: Adapted from the diabetes distress scale [21,22]. |  |
| Distress 1 | Overwhelmed with the demands of living with IBD? | 1. Not at all  2. Very little  3. Somewhat  4. Quite a bit  5. A great deal |
| Distress 2 | That you are often failing with your IBD routine? | 1. Not at all  2. Very little  3. Somewhat  4. Quite a bit  5. A great deal |
| Distress 3 | Discouraged to keep up with managing your IBD? | 1. Not at all  2. Very little  3. Somewhat  4. Quite a bit  5. A great deal |
| Distress 4 | Angry, scared, and/or depressed when thinking about living with IBD? | 1. Not at all  2. Very little  3. Somewhat  4. Quite a bit  5. A great deal |
| Distress 5 | That your friends of family don’t appreciate how difficult living with IBD can be? | 1. Not at all  2. Very little  3. Somewhat  4. Quite a bit  5. A great deal |
| Distress 6 | That your friends or family don’t give you the emotional support that you would like? | 1. Not at all  2. Very little  3. Somewhat  4. Quite a bit  5. A great deal |
| **Perceived IBD support** | | |
| General IBD support | When it comes to your IBD, how much support in general do you feel you have?   Source: Developed for this survey | 1. Not at all  2. A little  3. Some  4. A lot  5. A great deal |
| **Self-efficacy: Remission management** | | |
| Maintaining remission prompt | In the past 2 weeks, how confident were you in your ability to...  Source: Adapted from the IBD Self-Efficacy Scale [24]. |  |
| Maintaining remission 1 | manage your IBD? | 1. Not sure at all  ... 11. Totally sure |
| Maintaining remission 2 | keep your IBD in remission? | 1. Not sure at all  ... 11. Totally sure |
| Maintaining remission 3 | engage in self-care? (e.g., exercise, diet, rest) | 1. Not sure at all  ... 11. Totally sure |
| Maintaining remission 4 | engage in a stress management activity? | 1. Not sure at all  ... 11. Totally sure |
| Maintaining remission 5 | maintain your sense of well-being? | 1. Not sure at all  ... 11. Totally sure |
| **Self-efficacy: Stress and emotion management** | | |
| Stress/emotion prompt | In the past 2 weeks, how confident were you in your ability to...  Source: Adapted from the IBD Self-Efficacy Scale [24]. |  |
| Stress/emotion 1 | do something to reduce stress from IBD? | 1. Not sure at all  ... 11. Totally sure |
| Stress/emotion 2 | do something to reduce discouragement about IBD? | 1. Not sure at all  ... 11. Totally sure |
| Stress/emotion 3 | do something to reduce sadness from IBD? | 1. Not sure at all  ... 11. Totally sure |
| **Self-efficacy: Symptoms management** | | |
| Symptoms management prompt | In the past 2 weeks, how confident were you in your ability to...  Source: Adapted from the IBD Self-Efficacy Scale [24]. |  |
| Symptoms management 1 | reduce IBD symptoms? | 1. Not sure at all  ... 11. Totally sure |
| Symptoms management 2 | reduce discomfort or pain from IBD? | 1. Not sure at all  ... 11. Totally sure |
| Symptoms management 3 | decrease fatigue from IBD? | 1. Not sure at all  ... 11. Totally sure |
| **Self-efficacy: Medication management** | | |
| Mediation. prompt | In the past 2 weeks, how confident were you in your ability to...  Source: Adapted from the IBD Self-Efficacy Scale [24]. | Medication self-efficacy items only asked to participants currently taking daily oral medication. |
| Medication 1 | follow your IBD medication prescription? | 1. Not sure at all  ... 11. Totally sure |
| Medication 2 | take your IBD medication at instructed times? | 1. Not sure at all  ... 11. Totally sure |
| Medication 3 | take you IBD medication as directed to prevent flare-up? | 1. Not sure at all  ... 11. Totally sure |
| **Coping strategies** | | |
| Coping prompt | In the past 2 weeks, how much have you...  Source: Adapted from the IBD-COPE instrument [25]. |  |
| Coping 1 | used relaxation techniques to help with your stress? | 1. Not at all  2. Very little  3. Somewhat  4. Quite a bit  5. A great deal |
| Coping 2 | tried to think positively about your IBD? (ex: “it makes me a stronger person”) | 1. Not at all  2. Very little  3. Somewhat  4. Quite a bit  5. A great deal |
| Coping 3 | altered your diet in an attempt to improve your IBD? | 1. Not at all  2. Very little  3. Somewhat  4. Quite a bit  5. A great deal |
